# Supplementary material for: Using the Traditional Ex Vivo Whole Blood Model to Discriminate Bacteria by Their Inducible Host Responses
Source: Biomedicines. 2024 Mar 25;12(4):724. doi: 10.3390/biomedicines12040724 (PMC11047930; doi:10.3390/biomedicines12040724)
Supplement: Supplementary file 1 [file biomedicines-12-00724-s001.zip › biomedicines-2915732-supplementary.pdf]

Supplementary Table S1. Bacterial strains used throughout this study
Isolate specific information of the eight bacterial strains used in this study

Species	Strain	Source	References
Escherichia coli	ECOR26	Part of the ECOR collection, Human infant isolate, Commensal strain	Ref 40: Ochman and Selander 1984
Escherichia coli	GMB10	Non-clinical plant isolate (Spinach)	Ref 39: Meric et al. 2013
Escherichia coli	K12	Model laboratory strain<br>Descendent of a strain first isolated in 1922 from a stool sample	Ref 41: Bachmann 1972<br>Ref 42: Gray and Tatum 1944<br>Ref 43: Lederberg 2004
Escherichia coli	B	Laboratory strain from pathology laboratory, Singleton Hospital, UK<br>Descendent of a strain originally isolated in 1918 and named in 1942	Ref 44: Daegelen, 2009 and refs therein
Staphylococcus aureus	VAP39	An MRSA, Cause of ventilator-associated pneumonia, UK isolate	Ref 45: Conway Morris et al. 2009<br>Ref 46: Conway Morris et al. 2011<br>Ref 47: Wilkinson et al. 2012
Staphylococcus aureus	SH1000	Representative laboratory strain of S. aureus, UK isolate, highly virulent derivative of NCTC 8325	Ref 48: O'Neill 2010
Staphylococcus epidermidis	1457	Cause of central-venous catheter associated disease, Biofilm producer	Ref 49: Mack et al 1992
Staphylococcus epidermidis	RP62A	Isolated from a patient with intravascular catheter-associated infection, Biofilm producer	Ref 50: Christenesen, 1982<br>Ref 51: Gill et al. 2005

A)

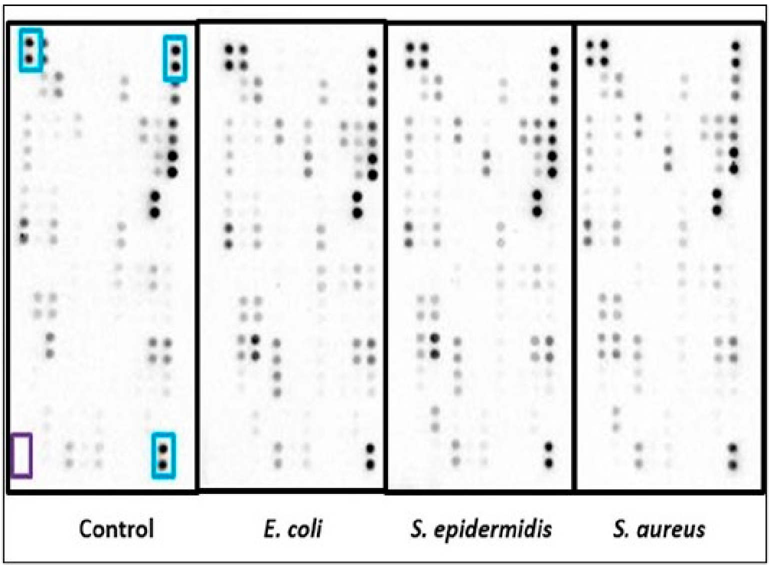

B)

Mediator	Control	K12	RP62A	SH1000
**Vitamin D BP**	2591.4	2162.8	3865	2390.4
**CD31**	4061.4	2911.8	2343.2	3505
**TIM-3**	2159.7	1947.6	1609.2	830.6
**VCAM-1**	10542.4	10108.2	8925.8	11297.5
**Serpin E1**	9492.6	12203.8	8952.4	15279.7
**SHBG**	3738.8	3448.3	2801.9	2709.5
**Thrombospondin-1**	3798.5	3781	3171.5	4171.2
**TNF-α**	538.5	5518.4	5565.1	4556.2
**Osteopontin**	6821.7	6069.3	4286.5	5304.3
**PF4**	4200.8	3851.7	3553.9	4983.1
**RANTES**	4482.5	3714.4	3750.8	3815.1
**RBP-4**	7358.2	16338	10638.7	6582
**Resistin**	1192.4	1195.4	3868.4	3139.8
**Lipocalin-2**	2343.4	5846.9	5298.5	5471.2
**MIP-1α**	10.6	6433.1	4111.4	3689.8
**MIP-3α**	162	8870.6	3308.7	1899.4
**MMP-9**	4348.7	4371.7	5330.9	5299.6
**IL-6**	83.3	2559.3	-4.5	886.1
**IL-8**	201.7	8591.9	6912.6	6982.3
**Growth Hormone**	2499.1	3165.4	2577.9	1911.5
**IGFBP-2**	4201.7	3569.2	3240	2969.1
**IGFBP-3**	2304.8	1776	1768.8	1661.3
**Endoglin**	5953.3	6250.3	4506	3707.7
**Chitinase 3-like 1**	24036.6	4459.2	9489.4	5380.3
**Complement factor D**	5828.8	4887.3	4549.8	3805.3
**C-reactive protein**	25379	19983.4	20952.2	9169.7
**Cystatin C**	3855	2736	2384.8	2424.7
**DPPIV**	7916.3	4351.9	4202.8	2969.5
**Adiponectin**	10784.1	7104.1	10800.9	8578.9
**Apolipoprotein A-I**	13614.1	9024	18278.4	10572.3
**Angiogenin**	22382.1	16179.4	20794.6	18757.9
**C5a**	7381.4	8614.3	8501	6704.1
**CD14**	2096.8	1562.5	1912.4	1306.1

Supplementary Figure S1. Bacterial induced cytokine screen for biomarker identification.
Upper panel A) Platelet-poor plasma from blood infected with *E. coli* K12, *S. epidermidis* 1457 or *S. aureus* SH1000, for 6 hours (n=1 donor) was added to cytokine array of 102 soluble mediators. The blue boxes indicate the six positive reference spots on a single membrane, and the purple box (bottom left) indicates the position of the two negative reference spots. Lower panel B); Quantitative analysis of the 33/102 cytokines that were detected on the cytokine array. All proteins where two spots could be detected on at least one (of the four) membranes were scanned using ImageJ and values were converted to pixels. Values represent the mean pixel density of two protein spots minus the mean of the negative control spots (purple square). Grey rows indicate >3-fold difference between the control membrane and at least one of the bacteria treated membranes.

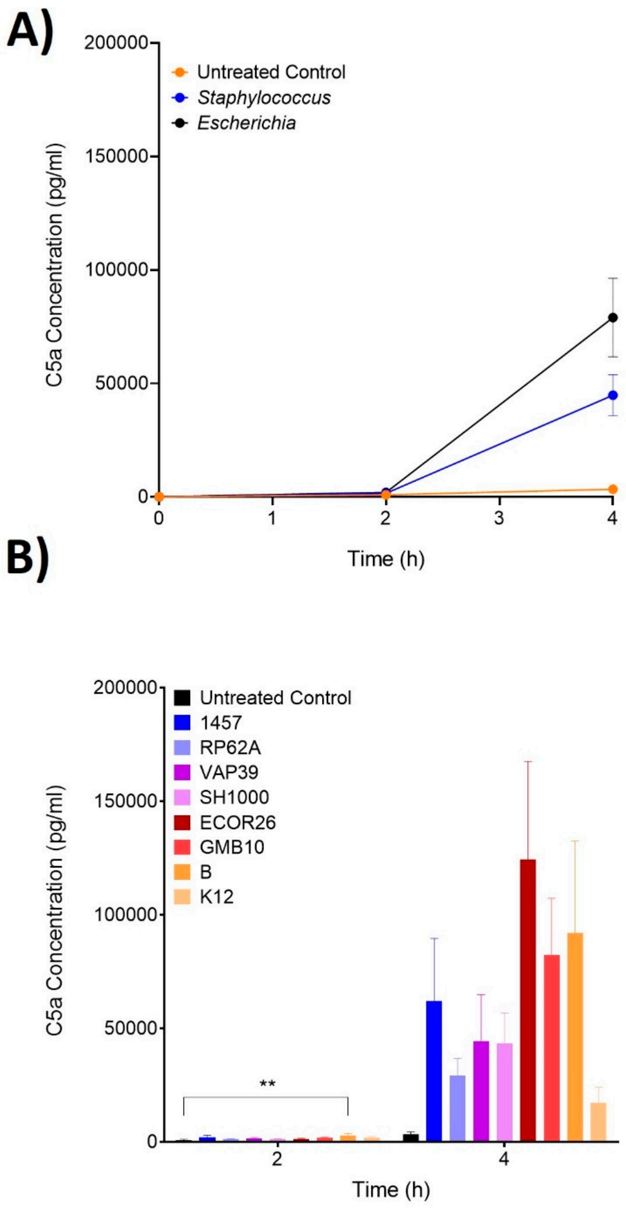

Supplementary Figure S2. Changes in C5a following *ex vivo* infection.
C5a was measured by ELISA after 2, 4 and 6 hours post-infection (n=5 donors). Plots show changes in C5a over time in response to A) Control, *Staphylococcus* and *Escherichia* genera and B) each strain at 2 and 4 hours (n = 5 donors). Error bars represent the mean ± SEM.

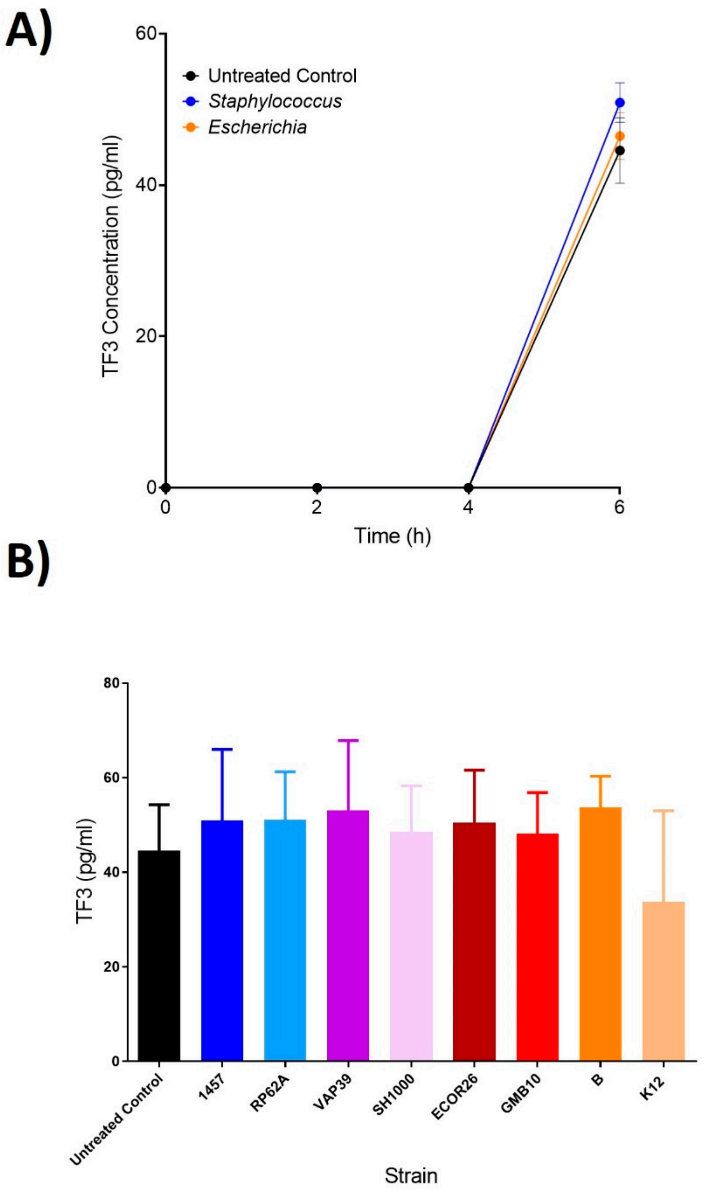

Supplementary Figure S3. Changes in TF3 following *ex vivo* infection.

TF3 was measured by ELISA after 2, 4 and 6 hours post-infection (n=5 donors). Plots show changes in C5a over time in response to A) Control, *Staphylococcus* and *Escherichia* genera and B) each strain at 6 hours (n = 5 donors). Error bars represent the mean ± SEM.

Supplementary Table S2. Linear Discriminant analysis of predicted and observed variables (strain level).
Data generated in the *ex vivo* models were analysed, including the 10 inflammatory outputs over three timepoints. Table S2 confirms the predicted and observed outcomes for strains.

	Observed (16/40, 40% correct)								
**Predicted**	Strain	B	E1457	Ecor26	GMB10	K12	RP62A	SH1000	VAP39
	B	4	0	0	0	1	0	0	0
	E1457	0	0	0	1	2	2	0	0
	Ecor26	1	0	2	2	0	0	0	0
	GMB10	0	0	2	2	0	0	0	1
	K12	2	1	1	1	0	0	0	0
	RP62A	0	0	0	1	0	4	0	0
	SH1000	0	0	0	0	0	2	3	0
	VAP39	1	0	0	0	2	0	1	1

Supplementary Table S3. Linear Discriminant analysis of predicted and observed variables (species level).
Data generated in the *ex vivo* models were analysed, including the 10 inflammatory outputs over three timepoints. Table S3 confirms the predicted and observed outcomes for species.

	Observed (36/40, 90% correct)			
**Predicted**	Species	*E.coli*	*S.aureus*	*S.epidermidis*
	E.coli	19	0	1
	S.aureus	0	8	2
	S.epidermidis	0	1	9

Supplementary Table S4. Linear Discriminant analysis of predicted and observed variables (genera level).
Data generated in the *ex vivo* models were analysed, including the 10 inflammatory outputs over three timepoints. Table S4 confirms the predicted and observed outcomes for genera.

	Observed		
**Predicted**	Genera	*Escherichia*	*Staphylococus*
	Escherichia	18	2
	Staphylococcus	0	20
